# lncRNA-DANCR Promotes Taxol Resistance of Prostate Cancer Cells through Modulating the miR-33b-5p-LDHA Axis

**DOI:** 10.1155/2022/9516774

**Published:** 2022-05-04

**Authors:** Yu-yong Wang, Chao Chen

**Affiliations:** Department of Urology, Affiliated Hangzhou First People's Hospital, Zhejiang University School of Medicine, Hangzhou, Zhejiang Province, China 310006

## Abstract

Prostate cancer (PCa) is one of the most common malignancies in men with high death rate worldwide. Paclitaxel (Taxol) is a widely used anticancer agent. Despite recent improvements in clinical application and research, development of drug resistance limits the efficacy of the Taxol-based chemotherapy. Previous studies revealed that the long noncoding RNA DANCR positively regulated progression of prostate cancer. However, the precise roles of DANCR in the Taxol sensitivity of PCa and the underlying molecular mechanisms remain largely unknown. Here, we report that the expressions of DANCR were significantly upregulated and miR-33b-5p were downregulated in prostate tumor specimens and cells as well as the Taxol-resistant prostate cancer cell line (PC3-TXR). Silencing DANCR or overexpressing miR-33b-5p effectively enhanced the Taxol sensitivity of PCa cells. Bioinformatics analysis, RNA pull-down assay, and luciferase assay consistently illustrated that DANCR was associated with miR-33b-5p, leading to downregulation of miR-33b-5p in PCa. Interestingly, glucose metabolism of PC3-TXR cells was remarkedly elevated. The glucose uptake, extracellular acidification rate (ECAR), and glycolysis speed-limiting enzyme expressions were significantly promoted in PC3-TXR cells. We further identified the glucose metabolism enzyme; LDHA was a direct target of miR-33b-5p in PCa cells. LDHA restoration attenuated miR-33b-5p-mediated PTX sensitization. Finally, the rescue of miR-33b-5p in DANCR-overexpressing PC3-TXR cells successfully overrode the DANCR-promoted Taxol resistance. In summary, this study uncovered biological roles and molecular mechanisms of the DANCR-promoted chemoresistance, contributing to the development of noncoding RNA-based therapeutic strategies against drug-resistant prostate cancer.

## 1. Introduction

Prostate cancer (PCa) is the most frequently diagnosed malignancy in men worldwide, resulting in poor prognosis and highly cancer-related deaths [1]. Currently, hormonal therapy (i.e., androgen deprivation) is a therapeutic approach by inducing antitumor response in prostate cancer patient [2]. In addition, chemotherapy and radiotherapy have been widely applied to reduce the risk of recurrence and improve overall survival of PCa patients at advanced or metastatic PCa [3]. Taxane (paclitaxel or docetaxel) is an antitumor drug through (a) inducing the polymerization of tubulin to stable microtubules and (b) directly associating with microtubules, resulting in cancer cell apoptosis [4, 5]. However, a large fraction of patients developed hormonal therapy resistance and Taxol resistance, resulting in tumor recurrence and metastasis [6]. Therefore, investigating the underlying cellular pathways and molecular targets of chemoresistant prostate cancer cells is an urgent task to prolong the overall survival rate of PCa patients.

Long noncoding RNAs (lncRNAs) are a group of noncoding RNAs which have relatively large size (>200 nucleotides) [7]. lncRNAs have been reported to play a vital role in prostate tumor progression including tumorigenesis, metabolism, proliferation, invasion, migration, metabolism, apoptosis, and chemoresistance [8, 9]. For instance, lncRNA NEAT1 was known to be frequently upregulated and functioned as a potential biomarker of prostate cancer [10]. In addition, SNHG17 has been reported to enhance the aggressiveness of human prostate cancer cells via modulating the *β*-catenin signaling [11]. lncRNA-DANCR (differentiation antagonistic nonprotein coding RNA) was highly expressed in diverse cancers and positively associated with tumor progressions [12]. Recent studies demonstrated that DANCR facilitated malignancy of prostate cancer by targeting miR-185-5p [13], suggesting that blocking the DANCR-mediated molecular signaling pathways may contribute to chemotherapy of PCa. However, the precise roles and molecular mechanisms of DANCR in Taxol-resistant PCa cells still remain elusive.

Accumulating studies uncovered that malignant tumors were characterized by the “Warburg effect,” which unveiled increased glucose uptake and lactic acid production of cancer cells instead of oxidative phosphorylation [14]. Moreover, blocking the dysregulated aerobic glycolysis could effectively inhibit cancer cell proliferation, migration, and chemoresistance [15]. Thus, understanding the precisely regulatory mechanisms of the anaerobic glycolysis of cancer cells will contribute to the development of effective anticancer strategies.

In this study, we aimed to investigate the roles of DANCR in glucose metabolism and Taxol resistance of prostate cancer. The DANCR-mediated ceRNA network as well as its molecular targets will be assessed. The effects of DANCR inhibition in overcoming Taxol resistance will be evaluated. Our results manifest critical roles of the DANCR-miR-33b-5p-LDHA axis in Taxol-resistant PCa cells, presenting a new insight for anti-chemoresistant PCa patient treatment.

## 2. Materials and Methods

### 2.1. Prostate Cancer Patient Tumor Tissue Collection

A total of 30 PCa patients were recruited in this study. Prostate normal tissue and tumor specimens were collected from the Affiliated Hangzhou First People's Hospital, Zhejiang University School of Medicine. Patients had undergone transrectal biopsies for histopathological diagnosis of PCa. The study was approved by the Ethics Committee of Affiliated Hangzhou First People's Hospital, Zhejiang University School of Medicine, in accordance with the Declaration of Helsinki. After dissection, the samples were frozen into liquid nitrogen immediately and stored at −80°C until use.

### 2.2. Cell Culture and Establishment of Taxol-Resistant Cells

The normal human prostate epithelial cells HPrEC, RWPE-1, and PCa cell lines (PC3, DU145, LN96, and OPCT-1) were purchased from American Tissue Culture Collection (Manassas, VA, USA). The cells were cultured in Dulbecco's Modified Eagle Medium (Gibco, Carlsbad, CA, USA) with 10% fetal bovine serum (Gibco) plus 1x penicillin and 1x streptomycin at 37°C under 5% CO_2_. The construction of PTX-resistant PCa cells (PC3-TXR) was conducted according to previous reports [16]. Briefly, PC3 cells were exposed to stepwise increased concentrations of PTX (Sigma, Shanghai, China) for two months. Survival clones were pooled for experiments of this study.

### 2.3. Cell Transfection

Prostate cancer cells were transfected with siRNA, miRNA, or vectors using the Lipofectamine 2000 (Invitrogen, Carlsbad, CA, USA) according to the manufacturer's instructions. siRNA against DANCR (siCCAT1), LDHA (siLDHA), and siRNA-negative control were synthesized by GenePharma (Shanghai, China). LDHA overexpression plasmid was purchased from http://Origene.com. miRNA-33b-5p and control miRNA were synthesized by GenePharma (Shanghai, China). siRNA and miRNA were transfected at 25 nM for 48 hours.

### 2.4. RNA Isolation and qRT-PCR

Total RNA was extracted from the tumor specimens and cells using TRIzol® (Thermo Fisher Scientific, Inc.). The quantity and quality of RNA samples were examined using a NanoDrop™ 2000/2000c Spectrophotometers (Thermo Fisher, Waltham, MA, USA). RNA was then reverse transcribed into cDNA using the SuperScript III reverse transcriptase kit (Thermo Fisher, Waltham, MA, USA) according to the manufacturer's protocols. The reaction of qRT-PCR was conducted using SYBR green master mix (Thermo Fisher, Waltham, MA, USA). Cycling conditions were 50°C for 2 min, 95°C for 10 min, then 95°C for 15 sec, and 60°C for 1 min for 40 cycles. PCR products were analyzed with the ABI PRISM 7900HT Sequence Detections System (Applied Biosystems; Thermo Fisher Scientific, Inc.). The relative mRNA level of the target gene was determined using ABI software (RQ Manager, version 1.2). The expressions of DANCR and LDHA were quantified to the housekeeping genes *β*-actin. The expressions of miR-33b-5p were quantified to human U6. The relative expression was calculated by the 2^−*ΔΔ*Ct^ method. Experiments were performed in triplicate and repeated three times.

### 2.5. Wound Healing Assay

PCa cell migration was assessed by wound healing assay. The cells (1 × 10^6^ cells/well) were seeded into 12-well plate for 24 hours. The cells were scratched a straight line using a P200 pipette tip. Medium was refreshed and cultured for 16 hours. Cell migration capacity was determined by the distance of migration. Experiments were performed in triplicate.

### 2.6. Colony Formation Assay

PCa cells were plated onto 12-well plates with a density of 500 cells/well after treating with different doses of Taxol and then cultured for 2 weeks. The cells were fixed with 4% PFA and then stained with 0.01% crystal violet (Sigma, Shanghai, China). The colony formation was analyzed under a bright-field microscope. Experiments were performed in triplicate.

### 2.7. Cell Viability Assay

Prostate cancer cells (1 × 10^4^ cells per well) were seeded into 96-well plates and then treated with different concentrations of PTX for 48 hours. Cell viability was determined by MTT (3-(4,5-dimethyl-2-thiazolyl)-2,5-diphenyl-2-H-tetrazolium bromide) (Sigma, Shanghai, China) assay according to the manufacturer's protocol. The absorbance was measured at 490 nm using a microplate reader (Bio-Rad, Hercules, CA, USA). Each experiment was performed in triplicate.

### 2.8. Cell Apoptosis Assay

Cell apoptosis rate was analyzed by flow cytometry using the Annexin V-(FITC)/propidium iodide (PI) apoptosis detection kit (BD Biosciences, San Jose, CA, USA) according to the manufacturer's instruction. After transfection and/or treatments, the cells were washed with PBS and then resuspended in binding buffer from this kit, followed by staining with 5 *μ*l Annexin V-FITC for 10 min and 5 *μ*l PI for 5 min in the dark. The analysis of cell apoptosis was carried out using a FACScan flow cytometer (BD Biosciences). Experiments were performed in triplicate and repeated three times.

### 2.9. Luciferase Assay

The putative binding sites of miR-33b-5p on DANCR or 3′UTR of LDHA were predicted by starBase [17]. The wild-type (WT) ormiR-33b-5p binding site mutant (Mut) DANCR or 3′-UTR sequences of LDHA were amplified and cloned into the pmirGLO vectors (Promega, Madison, WI, USA). PCa cells were cotransfected with control miRNA or miR-33b-5p with WT- or Mut-DANCR or 3′UTR of LDHA using Lipofectamine 2000 according to the manufacturer's protocols for 48 h. Luciferase activity was determined with a luciferase assay kit (Promega, Madison, WI, USA) according to the manufacturer's instructions. Experiments were performed in triplicate and repeated three times.

### 2.10. RNA Pull-Down Assay

The RNA-RNA interaction was assessed by RNA pull-down assay as previously described [18]. Scramble control and sense- and antisense-DANCR probes were biotin-labeled from the RiboBio Co. Ltd. (Guangzhou, China). Cell lysates from PCa were incubated with individual probe for 2 hours. Lysates were then incubated with Streptavidin-coupled agarose beads for 2 more hours. The amount of miR-33b-5p in the RNA-RNA complex was determined by qRT-PCR. Experiments were repeated three times.

### 2.11. Evaluation of the Glucose Metabolism

The glucose metabolism was measured by glucose uptake, ECAR, and glycolysis enzyme expressions. The glucose uptake was performed using the Glucose Uptake Colorimetric Assay Kit (#MAK083, Sigma-Aldrich, Shanghai, China), and the ECAR was measured using the Seahorse XF Glycolysis Stress Test Kit (Agilent, Santa Clara, CA, USA) according to the manufacturer's instructions. Experiments were performed in triplicate and repeated three times.

### 2.12. Western Blot Analysis

Prostate cancer cells treated with Taxol were collected and lysed with RIPA lysis buffer (Beyotime Biotech, Shanghai, China) plus protease inhibitor (Sigma, Shanghai, China) on ice for 15 min. Lysates were centrifuged at 12,000 g for 10 min and supernatant was collected. Protein samples were quantified using a Bradford assay kit (Thermo Fisher). Equal amounts of proteins were loaded onto and separated via SDS-PAGE gel electrophoresis. After the electrophoresis, proteins were transferred to PVDF (polyvinylidene fluoride) membranes (Millipore, Billerica, MA, USA) and blocked with 5% nonfat milk for 1 h at room temperature. Membranes were incubated with primary antibodies for overnight at 4°C followed by washing with PBS for 3 × 10 min. Membranes were interacted for 2 h at room temperature with horseradish peroxidase-conjugated secondary antibody. Protein bands were visualized using the enhanced chemiluminescence chromogenic substrate kit (Beyotime Biotech, Shanghai, China). Experiments were repeated three times.

### 2.13. Statistical Analysis

Statistical analysis was performed using the GraphPad Prism 7.0 software (La Jolla, CA, USA). Data were expressed as the mean ± standard deviation (S.D). Statistical analysis between two groups was conducted by Student's *t* test, and the comparison among multiple groups was analyzed using one-way analysis of variance (ANOVA). The linear correlation between DANCR, miR-33b-5p, and LDHA was analyzed using Spearman's correlation coefficient. *p* value < 0.05 was considered statistically significant.

## 3. Results

### 3.1. lncRNA-DANCR Is Upregulated in Prostate Cancer and Promotes Taxol Resistance

**Figure 1:**
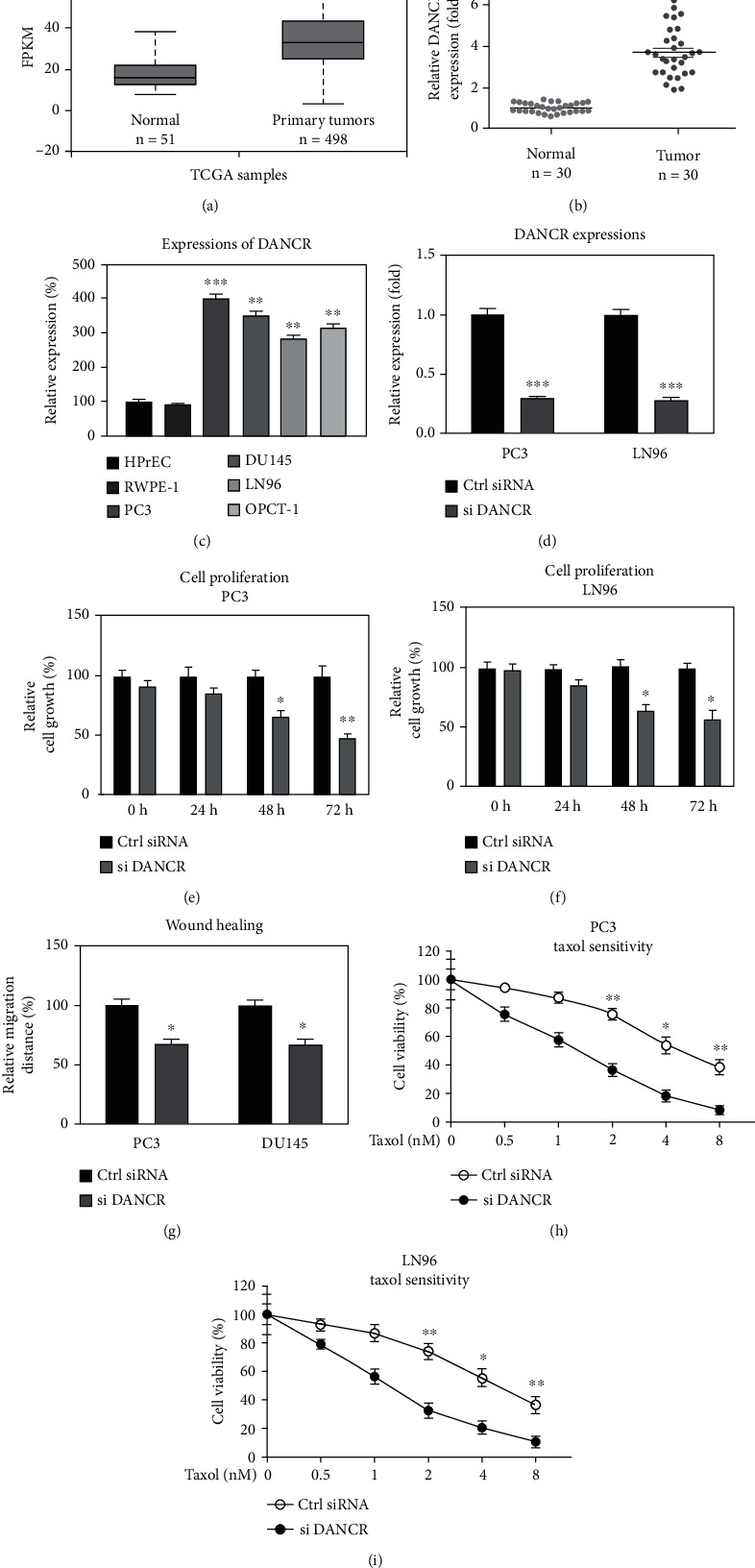
DANCR contributes to Taxol resistance of PCa. (a) Expressions of lncRNA-DANCR from prostate tumors and normal prostate tissues were analyzed by http://ualcan.path.uab.edu from TCGA database. (b) Expressions of lncRNA-DANCR from prostate tumors (*n* = 30) and adjacent normal tissues (*n* = 30) were analyzed by qRT-PCR. (c) Expressions of DANCR were examined in two non-tumorous prostate cell lines and four PCa cell lines. (d) PC3 and LN96 cells were transfected with control siRNA or DANCR siRNA for 48 hours. Expression of DANCR was detected. (e and f) The above cells were subjected to cell proliferation assay at 0, 24 h, 48 h, and 72 h by MTT assay. (g) Cell migration of the above transfected cells were examined by wound healing assay. (h and i) PC3 and LN96 cells without and with DANCR knockdown were treated with Taxol. Cell viability was examined by MTT assay. ^∗^*p* < 0.05, ^∗∗^*p* < 0.01, and ^∗∗∗^*p* < 0.001.

To evaluate the potential roles of DANCR in PCa, its abundance was compared in PCa specimens and corresponding normal prostate tissues. Bioinformatics analysis showed that the expression of DANCR was significantly elevated in diverse cancers particularly in prostate cancer from TCGA cancer database (Figure 1(a) and Fig. S1A and S1B). Expectedly, the expression of DANCR was significantly enhanced in PCa tissues by qRT-PCR (Figure 1(b)). Similarly, high expressions of DANCR were also detected in four prostate cancer cell lines compared with those from two normal prostate cell lines (Figure 1(c)). To investigate the roles of DANCR in PCa progression and chemoresistance, DANCR was knockdown by siRNA in PC3 and LN96 cells (Figure 1(d)). Cell viability assay showed that PCa cells with low DANCR expression exhibited retarded growth rate at 48 and 72 hours (Figures 1(e) and 1(f)). Silencing DNACR-rendered PCa cells suppressed migration from *in vitro* wound healing assay (Figure 1(g)). Moreover, with low DANCR expressions, PCa cells were more sensitive to Taxol treatments (Figures 1(h) and 1(i)). The Taxol IC50s of PC3 and LN96 cells were 5.73 nM and 4.52 nM. The inhibition of DANCR effectively reduced the IC50s of PCa cells to 1.26 nM and 1.47 nM, respectively. Summarily, these results indicated that DANCR contributes to proliferation, migration, and Taxol resistance of prostate cancer cells.

### 3.2. Taxol-Resistant PCa Cells Display Elevated DANCR Expression

**Figure 2:**
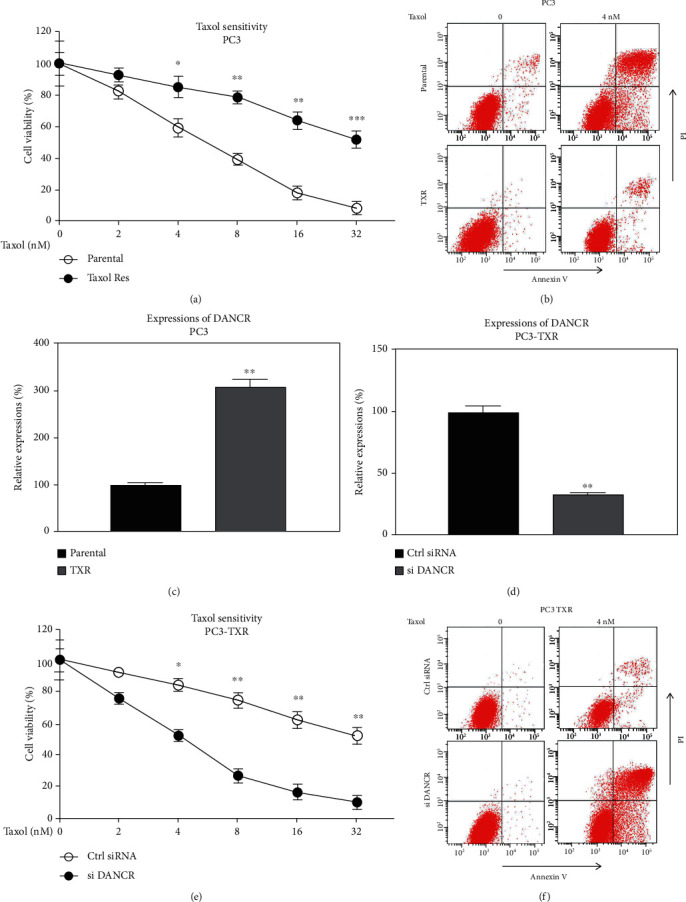
Taxol-resistant cells display elevated DANCR expression. (a) Parental and Taxol-resistant PC3 cells were treated with Taxol at the indicated concentrations for 48 hours. The Taxol responses were examined by MTT assay or (b) Annexin V apoptosis assay. (c) Expressions of DANCR in parental and Taxol-resistant PC3 cells. (d) PC3-TXR cells were transfected with control siRNA or DNACR siRNA; the expressions of DANCR were determined. (e) The above transfected PC3-TXR cells were treated with Taxol at the indicated concentrations; cell survival was examined by MTT assay and (f) Annexin V apoptosis assay. ^∗^*p* < 0.05, ^∗∗^*p* < 0.01, and ^∗∗∗^*p* < 0.001.

To investigate the functions of DANCR in Taxol resistance, the PTX-resistant PCa cell line (PC3-TXR) was established by exposing PC3 parental cells with gradually elevated concentrations of Taxol. The results showed that compared with parental cells, PC3-TXR cells could withstand higher concentrations of TXR treatments from cell viability assay and apoptosis assay (Figures 2(a) and 2(b)). The TXR IC50 of PC3-TXR cells was increased to 33.65 nM, which is around 6 folds of that from PC3 parental cells. We observed higher level of DANCR in TXR cells compared with parental cells (Figure 2(c)). To explore whether targeting DANCR could resensitize PC3-TXR cells to Taxol, DANCR was silenced in PC3 parental and TXR cells (Figure 2(d)). As we expected, silencing DANCR effectively sensitized TXR cells to Taxol (Figures 2(e) and 2(f)). The IC50 of TXR cells with low DANCR dropped to 7.22 nM compared with that from control cells (34.02 nM). Taken together, the above results demonstrate that blocking DANCR could effectively resensitize Taxol-resistant PCa cells to Taxol.

### 3.3. DANCR Sponges miR-33b-5p to Downregulate Its Expression in PC Cells

**Figure 3:**
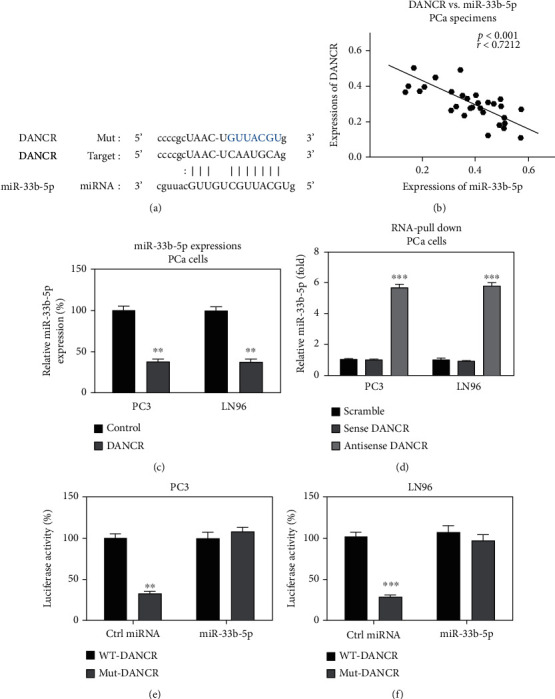
DANCR sponges miR-33b-5p in PCa. (a) Prediction of the DANCR-miR-33b-5p association from starBase. (b) Correlation analysis of DANCR and miR-33b-5p in prostate tumor specimens. (c) PC3 and LN96 cells were transfected with control siRNA or DNACR siRNA; the expression of miR-33b-5p was examined. (d) RNA-pull down assay was performed using lysates from PCa cells and sense or antisense DANCR probe. (e and f) Luciferase assay was performed in PC3 and LN96 cells cotransfected with control miRNA or miR-33b-5p plus WT-DANCR or Mut-DANCR luciferase vector. ^∗∗^*p* < 0.01 and ^∗∗∗^*p* < 0.001.

We then investigated the underlying mechanisms of the DANCR-promoted Taxol resistance in prostate cancer cells. Given studies have revealed that lncRNAs sponge target miRNAs through a ceRNA network, resulting in upregulation of target mRNAs of miRNAs [19]; bioinformatics analysis was performed to seek the potential DANCR targets through noncoding RNA database. Interestingly, miRNA-33b-5p, which was known as a tumor suppressive miRNA in diverse cancers [20, 21], was observed to contain putatively DNACR binding sites (Figure 3(a)). Moreover, a significantly negative correlation between DANCR and miR-33b-5p in PCa specimens was detected by Pearson's correlation coefficient analysis (Figure 3(b)). To test whether DANCR could downregulate miR-33b-5p expression, DANCR was overexpressed in PC3 and LN96 cells. Expected results demonstrated that PC cells with higher DANCR expressions accompanied lower miR-33b-5p expressions (Figure 3(c)). To verify the association between DANCR and miR-33b-5p, RNA-pull down assay was performed. The results showed that miR-33b-5p was associated with antisense DANCR probe detected by qRT-PCR (Figure 3(d)), while miR-33b-5p could not be effectively precipitated by scramble control or DANCR sense probe (Figure 3(d)). Furthermore, luciferase assay was performed, and the results demonstrated that PC3 and LN96 cells which were cotransfected with miR-33b-5p plus luciferase vector containing WT-DANCR displayed significantly inhibited luciferase activities (Figures 3(e) and 3(f)). Luciferase activities of the cells transfected with control miRNA plus WT-DANCR or miR-33b-5p plus predicted binding site mutant DANCR (Mut-DANCR) were not affected (Figure 3(e)). Summarily, the above results validated that DANCR sponges miR-33b-5p to inhibit expression in prostate cancer cells.

### 3.4. miR-33b-5p Is Negatively Associated with Prostate Cancer and Taxol Resistance

**Figure 4:**
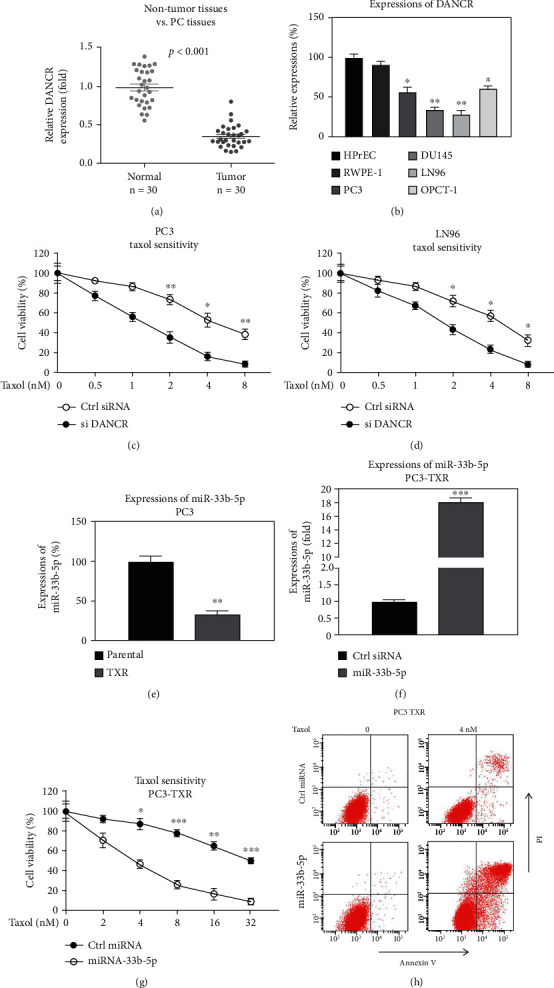
miR-33b-5p is negatively associated with Taxol resistance. (a) Expressions of miR-33b-5p from prostate tumors (*n* = 30) and adjacent normal tissues (*n* = 30) were analyzed by qRT-PCR. (b) Expressions of miR-33b-5p were examined in two non-tumorous prostate cell lines and four PCa cell lines. (c and d) PC3 and LN96 cells were transfected with control miRNA or miR-33b-5p for 48 hours. Cells were treated with Taxol at the indicated concentrations. Cell viability was examined by MTT assay. (e) Expressions of miR-33b-5p were examined in parental and Taxol-resistant PC3 cells. (f) PC3-TXR cells were transfected with control miRNA or miR-33b-5p; the expressions of miR-33b-5p were shown. (g) The above cells were treated with Taxol at the indicated concentrations; cell survival was examined by MTT assay and (h) Annexin V apoptosis assay. ^∗^*p* < 0.05, ^∗∗^*p* < 0.01, and ^∗∗∗^*p* < 0.001.

Given the above-described oncogenic roles of DANCR and the DANCR-miR-33b-5p ceRNA network in PCa, we therefore hypothesized miR-33b-5p functions as a tumor suppressor in prostate cancer cells. We detected that miR-33b-5p was significantly downregulated in prostate tumor specimens compared with their adjacent nontumorous prostate tissues (Figure 4(a)). Consistently, miR-33b-5p was remarkedly suppressed in prostate cancer cell lines compared with normal prostate cells (Figure 4(b)). To test the potential role of miR-33b-5p in Taxol sensitivity, miR-33b-5p was overexpressed in prostate cancer cells PC3 and LN96. Expectedly, PCa cells with overexpression of miR-33b-5p displayed significantly increased Taxol sensitivity compared with control cells (Figures 4(c) and 4(d)). Furthermore, miR-33b-5p was remarkedly downregulated in PC3-TXR cells (Figure 4(e)). The overexpression of miR-33b-5p (Figure 4(f)) effectively resensitized PC3-TXR cells to Taxol (Figures 4(g) and 4(h)). In summary, these results conclude that miR-33b-5p is negatively associated with Taxol resistance of prostate cancer cells.

### 3.5. Glucose Metabolism Is Elevated in Taxol-Resistant PCa Cells

**Figure 5:**
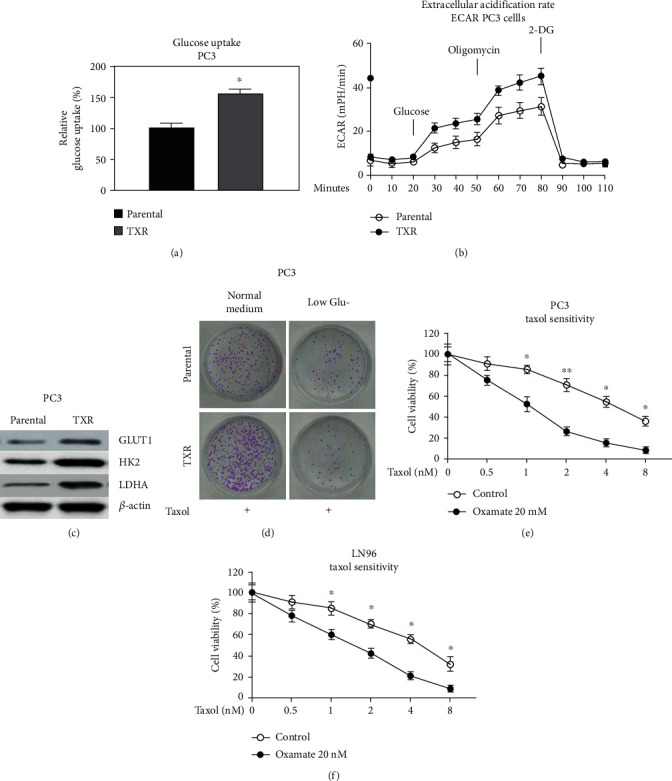
Glucose metabolism is elevated in Taxol-resistant prostate cancer cells. (a) Glucose uptake and (b) ECAR were examined in PC3 parental and TXR cells. (c) Glucose metabolism enzymes were examined by Western blot in PC3 parental and TXR cells. (d) PC3 parental and TXR cells were cultured with normal or low glucose medium for 48 hours, followed by treatment with Taxol. Survival cells were examined by clonogenic assay. (e and f) PC3 and LN96 cells were cotreated with Taxol plus glycolysis inhibitor, oxamate, at the indicated concentrations, and cell viability was determined by MTT assay. ^∗^*p* < 0.05 and ^∗∗^*p* < 0.01.

The cellular and molecular mechanisms of the noncoding RNA network-regulated Taxol resistance were consequently investigated. Accumulation studies revealed that dysregulated glucose metabolism influenced chemosensitivity of cancer cells [14, 15]; we evaluated the roles of DANCR in glucose metabolism of PCa cells. Expectedly, the overall glucose consumption and the extracellular acidification rate (ECAR), two readouts for glucose metabolism, were remarkedly increased in PC3-TXR cells (Figures 5(a) and 5(b)). In addition, the glucose metabolism key enzymes, GLUT1, HK2, and LDHA, were significantly upregulated in Taxol-resistant cells (Figure 5(c)). Consistently, under low glucose supply, Taxol treatments were more efficient in PC3-TXR cells than parental cells (Figure 5(d)). To test whether targeting glycolysis will contribute to enhancement of anticancer effects of Taxol, PC3 and LN96 cells were cotreated with Taxol plus glycolysis inhibitor, oxamate. Expected results showed that cotreatment of oxamate significantly increased the Taxol sensitivities of PC3 and LN96 cells from cell viability assay (Figures 5(e) and 5(f)). Taken together, these results consistently uncovered an effective approach against Taxol resistance of PCa cells by blocking glucose metabolism.

### 3.6. DANCR and miR-33b-5p Invertedly Regulate Glucose Metabolism of Prostate Cancer Cells

**Figure 6:**
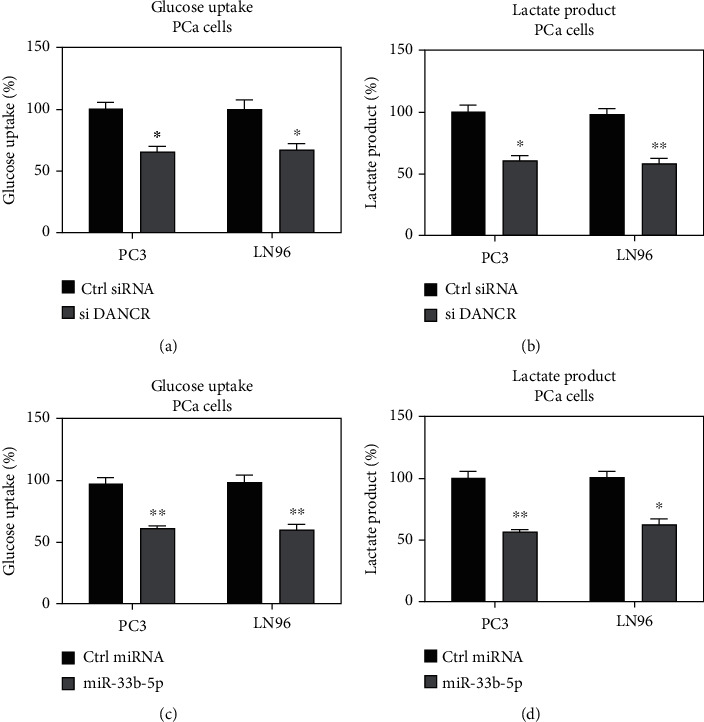
DANCR and miR-33b-5p reversely regulate glucose metabolism. (a) PC3 and LN96 cells were transfected with control siRNA or DANCR siRNA, and the glucose uptake and (b) lactate product were detected. (c) PC3 and LN96 cells were transfected with control miRNA or miR-33b-5p, and the glucose uptake and (d) lactate product were detected. ^∗^*p* < 0.05 and ^∗∗^*p* < 0.01.

Since a negative correlation was detected between DANCR and miR-33b-5p in Taxol resistance, we then evaluated the roles of the DANCR-miR-33b-5p ceRNA in glucose metabolism. DANCR was silenced in PC3-TXR cells. The glucose uptake (Figure 6(a)) and lactate product (Figure 6(b)) were significantly suppressed in DANCR-knockdown cells. On the other way, PC3-TXR cells with overexpression of miR-33b-5p displayed similar glycolysis phenotypes (Figures 6(c) and 6(d)), indicating DNACR sponges miR-33b-5p to promote glycolysis of Taxol-resistant PCa cells.

### 3.7. miR-33b-5p Directly Targets LDHA to Sensitize PCa Cells to Taxol

**Figure 7:**
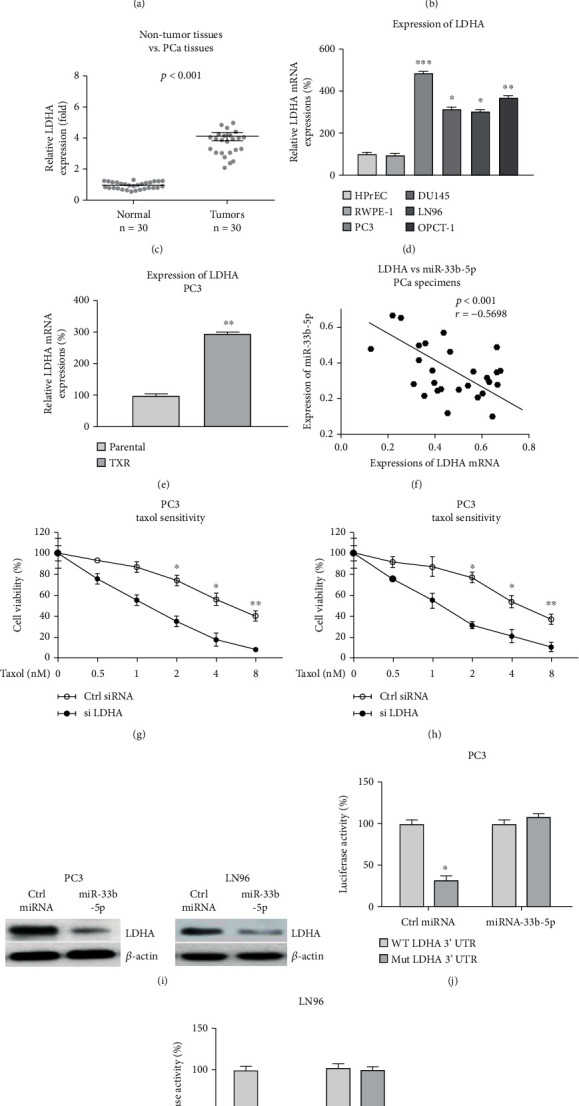
miR-33b-5p targets 3′UTR of LDHA in PCa cells. (a) Prediction of binding sites of miR-33b-5p on LDHA 3′UTR. (b) Expressions of LDHA from prostate tumors and normal prostate tissues were analyzed by http://ualcan.path.uab.edu from TCGA database. (c) Expressions of miR-LDHA from prostate tumors (*n* = 30) and adjacent normal tissues (*n* = 30) were analyzed by qRT-PCR. (d) Expressions of LDHA were examined in two non-tumorous prostate cell lines and four PCa cell lines. (e) Expressions of LDHA were examined in parental and Taxol-resistant PC3 cells. (f) Correlation analysis of LDHA and miR-33b-5p in prostate tumor specimens. (g and h) PC3 and LN96 cells were transfected with control miRNA or miR-33b-5b. Cells were treated with Taxol, and cell viability was determined by MTT assay. (i) Protein expressions of LDHA were examined from the above transfected cells. (j and k) Luciferase assay was performed in PC3 and LN96 cells with cotransfection of control miRNA or miR-33b-5p plus luciferase vector containing WT- or Mut-LDHA 3′UTR. ^∗^*p* < 0.05, ^∗∗^*p* < 0.01, and ^∗∗∗^*p* < 0.001.

It was known that miRNAs bind to the 3′UTR regions of their target mRNAs, resulting in impairing the mRNA stability and translation [19]. The mRNA targets of miR-33b-5p were predicted from noncoding RNA database. Interestingly, the 3′UTR of LDHA, which catalyzes the conversion of pyruvate to lactate, the speed-limit reaction during glucose metabolism [22], contains putative miR-33b-5p binding sites (Figure 7(a)). The clinical relevance of LDHA in prostate cancer was assessed. LDHA was significantly upregulated in PCa tumor specimens compared with normal prostate tissues by bioinformatics analysis from TCGA cancer database and qRT-PCR (Fig. S2 and Figures 7(b) and 7(c)). In addition, PCa patients with higher LDHA expressions displayed relative lower survival rates (Fig. S3). Consistently, LDHA was significantly upregulated in PCa cell lines and Taxol-resistant cells compared with normal prostate cells and parental PCa cells, respectively (Figures 7(d) and 7(e)). Pearson's correlation coefficient analysis illustrated that lower miR-33b-5p expression was significantly associated with higher LDHA expression in PCa tumor specimens (Figure 7(f)), suggesting that miR-33b-5p could target LDHA in prostate cancer. To verify the functions of LDHA in Taxol sensitivity of PCa cells, LDHA was knocked down by siRNA in PC3 and LN96 cells. Expectedly, PCa cells with LDHA silencing displayed significant sensitization to Taxol treatments compared with control cells (Figures 7(g) and 7(h)). We then investigated whether miR-33b-5p could target LDHA in PCa cells. The overexpression of miR-33b-5p significantly blocked LDHA protein expressions in PCa cells (Figure 7(i)). To further validate whether miR-33b-5p could directly target the 3′UTR of LDHA, luciferase reporter assays were performed. The results from luciferase assay demonstrated that cotransfection of luciferase vector containing wild-type 3′UTR of LDHA with miR-33b-5p effectively suppressed the luciferase activity (Figures 7(j) and 7(k)), while cotransfection of binding site mutant 3′UTR of LDHA with miR-33b-5p could not affect the luciferase activity. Summarily, these results consistently validated miR-33b-5p block LDHA expression through direct targeting 3′UTR of LDHA mRNA.

**Figure 8:**
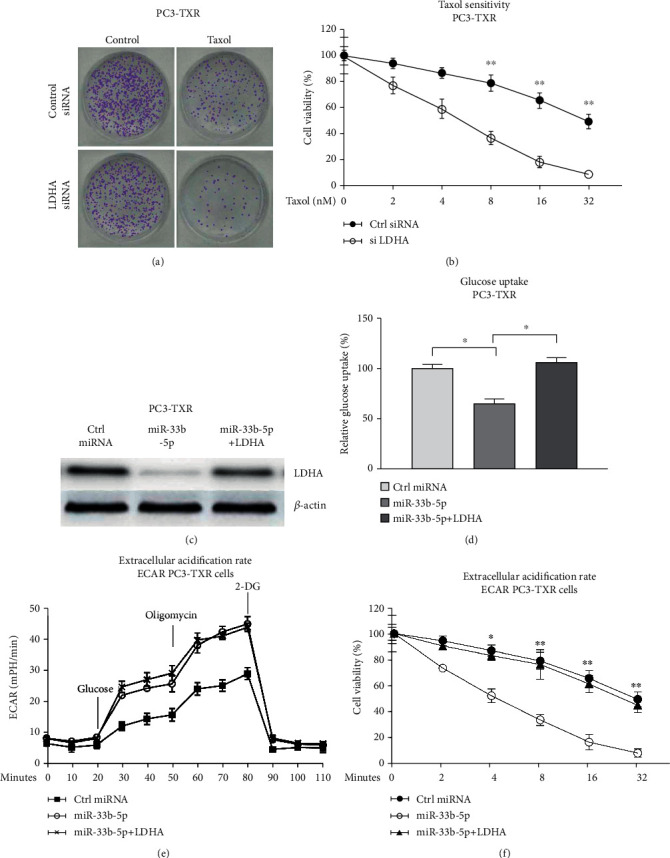
Restoration of LDHA rescues the miR-33b-5p-promoted Taxol sensitization. (a) PC3-TXR cells without or with LDHA knockdown were treated with Taxol. Cell survival was evaluated by clonogenic assay and (b) MTT assay. (c) PC3-TXR cells were transfected with control miRNA, miR-33b-5p alone, or plus LDHA overexpression plasmid. Protein expression of LDHA was determined. (d) Glucose uptake and (e) lactate product from the above transfected cells were examined. (f) The above transfected PC3-TXR cells were treated with Taxol at the indicated concentrations. Cell viability was determined by MTT assay. ^∗^*p* < 0.05 and ^∗∗^*p* < 0.01.

Rescue experiments were performed to validate whether the miR-33b-5p-supressed glucose metabolism and Taxol resistance were through targeting LDHA. PC3-TXR cells with LDHA knockdown displayed higher Taxol sensitivity compared with control siRNA-transfected cells (Figures 8(a) and 8(b)), suggesting that blocking LDHA/glycolysis could resensitize TXR cells to Taxol. Furthermore, cotransfection of LDHA with miR-33b-5p successfully rescued LDHA protein expression (Figure 8(c)). Expectedly, recovery of LDHA in miR-33b-5p-overexpressing TXR cells significantly restored the glucose uptake (Figure 8(d)) and ECAR (Figure 8(e)) in PC3-TXR cells. Importantly, PC3-TXR cells with rescue of LDHA displayed recovered Taxol resistance compared with miR-33b-5p overexpress alone cells (Figure 8(f)). In summary, the rescue experiments verified that miR-33b-5p sensitizes TXR cells by targeting LDHA/glucose metabolism.

### 3.8. DANCR Promotes Taxol Resistance through Modulating the miR-33b-5p-LDHA Axis in PCa Cells

**Figure 9:**
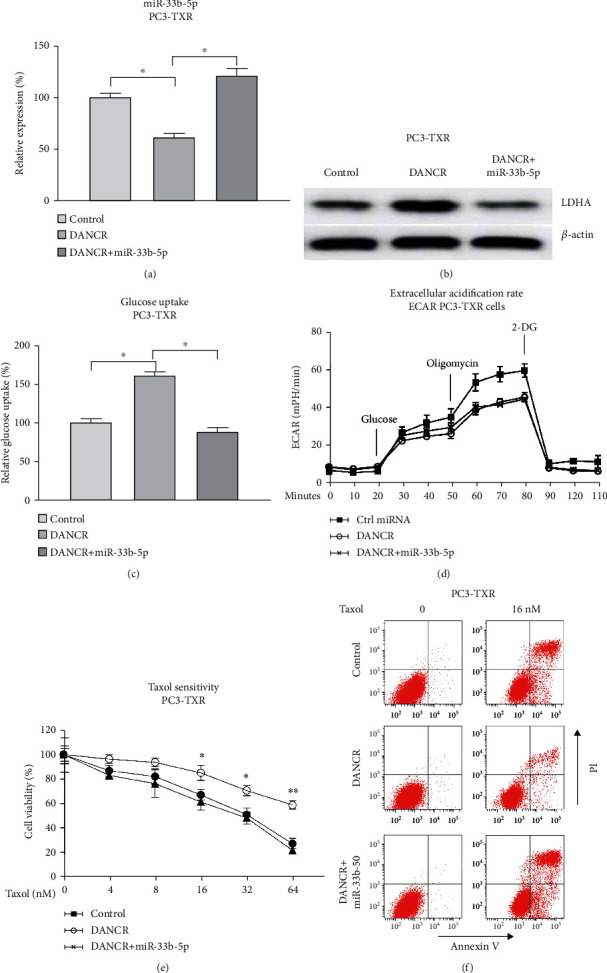
The roles of DANCR-miR-33b-5p-LDHA in Taxol resistance of PCa cells. (a) Control vector, DANCR alone, or plus miR-33b-5p was transfected into PC3-TXR cells. Expressions of miR-33b-5p, (b) LDHA, (c) glucose uptake, and (d) lactate product were detected. (e) The above transfected cells were treated with Taxol at the indicated concentrations. Cell survival was evaluated by MTT assay and (f) Annexin V apoptosis assay. ^∗^*p* < 0.05 and ^∗∗^*p* < 0.01.

Finally, we evaluated whether the DANCR-promoted Taxol resistance was through the miR-33b-5p-LDHA/glycolysis axis. Mechanism rescue experiments were performed by cotransfection of control vector, DANCR alone, or plus miR-33b-5p into PC3-TXR cells. qRT-PCR and Western blot results demonstrated that the overexpression of DANCR significantly blocked miR-33b-5p and upregulated LDHA expression, which were further reversed by rescue of miR-33b-5p (Figures 9(a) and 9(b)). Expectedly, cotransfection of DANCR with miR-33b-5p recovered the glucose uptake (Figure 9(c)) and ECAR (Figure 9(d)) of PC3-TXR cells. Expectedly, under Taxol treatments, significant recovery of Taxol sensitivity in miR-33b-5p-restored cells was observed compared with that from DANCR overexpression cells (Figures 9(e) and 9(f)). Taken together, our results concluded that the DANCR-modulated Taxol resistance in PCa cells was through targeting the miR-33b-5p-LDHA/glucose metabolism axis.

## 4. Discussion

Prostate cancer remains the most frequently diagnosed malignancy in men, leading to pool prognosis and highly cancer-related deaths [1]. Hormonal therapy, chemotherapy, and radiotherapy have been applied to reduce the risk of recurrence and improve overall survival of PCa patients at advanced or metastatic PCa [2]. Despite the initially effective response, a large number of PCa patients developed drug resistance. This study was aimed at investigating the biological functions and mechanisms for the lncRNA-DANCR-mediated Taxol sensitivity of PCa cells. We observed that DANCR was significantly upregulated in PCa patient tissues and cell lines, indicating that DANCR is positively associated with progression of prostate cancer. Moreover, silencing DNACR effectively suppressed the growth and Taxol resistance of PCa cells. Furthermore, from the selected Taxol-resistant PCa cell line, DANCR was detected to be remarkedly upregulated in PC3-TXR cells compared with parental cells. Although studies have described that higher DANCR expression was positively correlated with diverse cancers [12, 13], the roles of the aberrant upregulation of DANCR in Taxol-resistant prostate cancers remain largely unknown. Our results consistently demonstrated that DANCR contributed to Taxol resistance of PCa, indicating that targeting DANCR might be applied as an anti-chemoresistance approach against prostate cancer.

Accumulating studies uncovered that noncoding RNAs are critical regulators for the tumorigenesis and progression of prostate cancer [8, 9]. miR-33b-5p was a known tumor suppressive miRNA which was negatively associated with diverse cancers such as renal cell carcinoma [21], gastric cancer [23], and osteosarcoma [24]. In this study, we demonstrated that miR-33b-5p was significantly downregulated in PCa specimens and cell lines as well as Taxol-resistant prostate cancer cells. The overexpression of miR-33b-5p effectively overcame Taxol resistance. Furthermore, since studies revealed that lncRNAs sponge miRNAs to alleviate their expressions, resulting in recovery of the miRNA-targeted mRNAs [19], we evaluated the DANCR-miR-33b-5b competing endogenous RNA network. By luciferase assay and RNA pull-down assay, we detected direct binding of miR-33b-5p on DANCR in prostate cancer cells. The results from the present study suggested that the DANCR-miR-33b-5p axis could be a critical biomarker and therapeutic target of prostate cancer.

Cancer cells, in contrast to untransformed cells, preferred catalyzing glucose through anaerobic glycolysis pathway but not mitochondrial respiration pathway [14]. This phenomenon is called “Warburg effect” [14]. Moreover, reprogramming of metabolism contributes to chemoresistance of cancer cells [15]. Cancer cells frequently display a glucose addiction phenotype, which is a potential target for anticancer treatments. Consistent with previous reports, we found that TXR PCa cells exhibited apparently elevated glucose metabolism rate. Silencing DANCR or overexpression of miR-33b-5p effectively suppressed glucose uptake and ECAR, consistent with the above uncovered biological roles of them in PCa. We investigated the mRNA targets of miR-33b-5p. Bioinformatics analysis predicted that LDHA, which is a speed-limiting enzyme in glycolysis, is a potential target of miR-33b-5p. Consequently, Western blot results and luciferase assay consistently validated that miR-33b-5p effectively bond to 3′UTR region of LDHA to downregulate its expression in prostate cancer cells. Furthermore, rescue experiments demonstrated that recovery of miR-33b-5p in DANCR-overexpressing PC3-TXR cells effectively overrode the Taxol resistance.

In summary, this study integrated the DANCR-miR-33b-5p-mediated Taxol resistance with the noncoding RNA-regulated glucose metabolism of PCa. Future experiments will focus on the validation of the above *in vitro* molecular mechanisms using a xenograft mice model. Our conclusions will provide a noncoding RNA-based molecular axis for the development of effectively anti-chemoresistant approaches for PCa treatment.

## Data Availability

The datasets used and/or analyzed during the current study are available from the corresponding author on reasonable request.

## Supplementary Materials

Supplementary 1 Figure S1: analysis of the expressions of DANCR from http://ualcan.path.uab.edu. (A) Expression of DANCR across TCGA tumors. (B) Expression of DANCR across TCGA cancers (with tumor and normal samples).

Supplementary 2 Figure S2: expression of LDHA across TCGA cancers (with tumor and normal samples) analyzed from http://ualcan.path.uab.edu.

Supplementary 3 Figure S3: effect of LDHA expression level on PRAD patient survival analyzed by the Kaplan-Meier plotter from https://kmplot.com/analysis/.
