# Supplementary material for: lncRNA-DANCR Promotes Taxol Resistance of Prostate Cancer Cells through Modulating the miR-33b-5p-LDHA Axis
Source: Dis Markers. 2022 May 4;2022:9516774. doi: 10.1155/2022/9516774 (PMC9096572; doi:10.1155/2022/9516774)
Supplement: Supplementary 1 — Figure S1: analysis of the expressions of DANCR from http://ualcan.path.uab.edu. (A) Expression of DANCR across TCGA tumors. (B) Expression of DANCR across TCGA cancers (with tumor and normal samples). [file 9516774.f1.pdf]

A

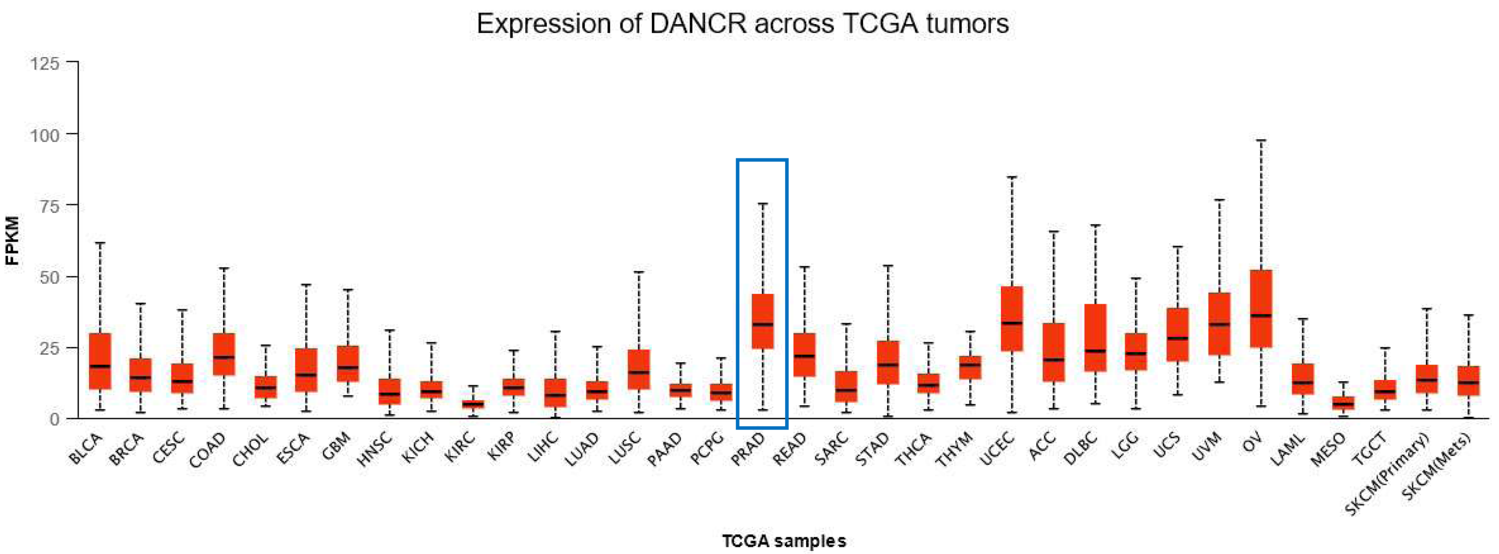
Expression of DANCR across TCGA tumors
FPKM
125
100
75
50
25
0
BLCA
BRCA
CESC
COAD
CHOL
ESCA
GBM
HNSC
KICH
KIRC
KIRP
LIHC
LUAD
LUSC
PAAD
PCPG
PRAD
READ
SARC
STAD
THCA
THYM
UCEC
ACC
DLBC
LGG
UCS
UVM
OV
LAML
MESO
TGCT
SKCM(Primary)
SKCM(Mets)
TCGA samples

B

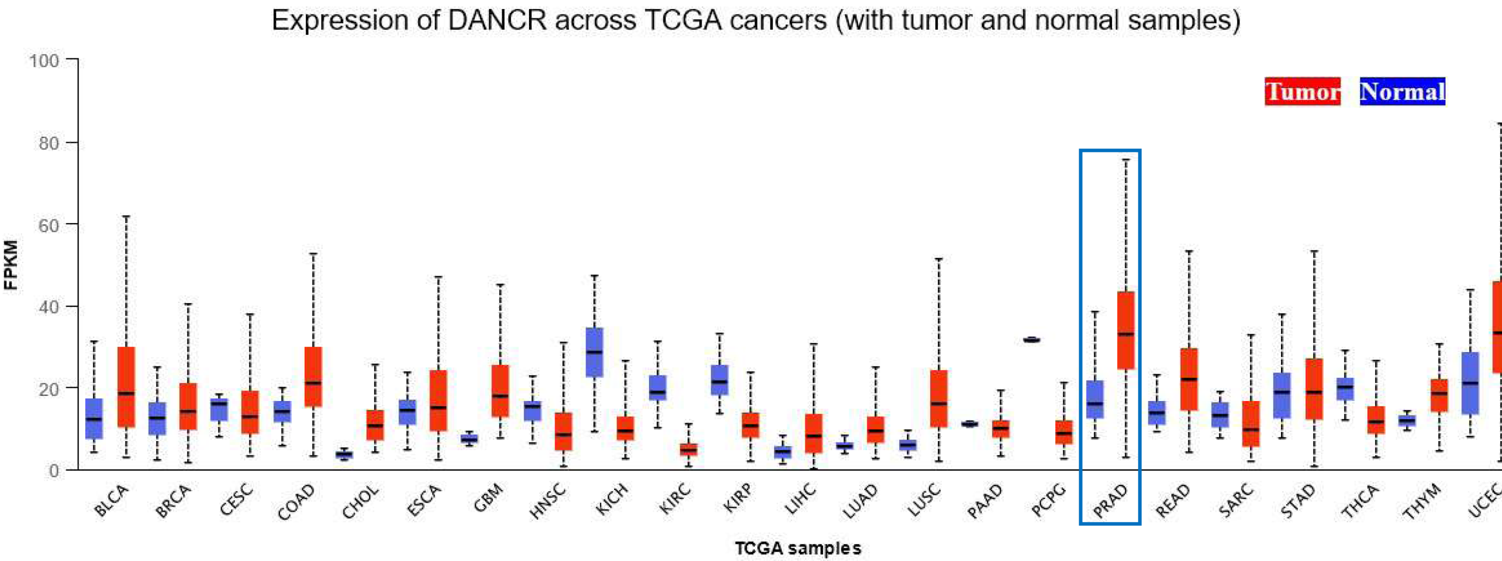
Expression of DANCR across TCGA cancers (with tumor and normal samples)
FPKM
100
80
60
40
20
0
Tumor
Normal
BLCA
BRCA
CESC
COAD
CHOL
ESCA
GBM
HNSC
KICH
KIRC
KIRP
LIHC
LUAD
LUSC
PAAD
PCPG
PRAD
READ
SARC
STAD
THCA
THYM
UCEC
TCGA samples
