# Supplementary figures and images for: lncRNA-DANCR Promotes Taxol Resistance of Prostate Cancer Cells through Modulating the miR-33b-5p-LDHA Axis
Source: Dis Markers. 2022 May 4;2022:9516774. doi: 10.1155/2022/9516774 (PMC9096572; doi:10.1155/2022/9516774)

Expression of LDHA across TCGA cancers (with tumor and normal samples)

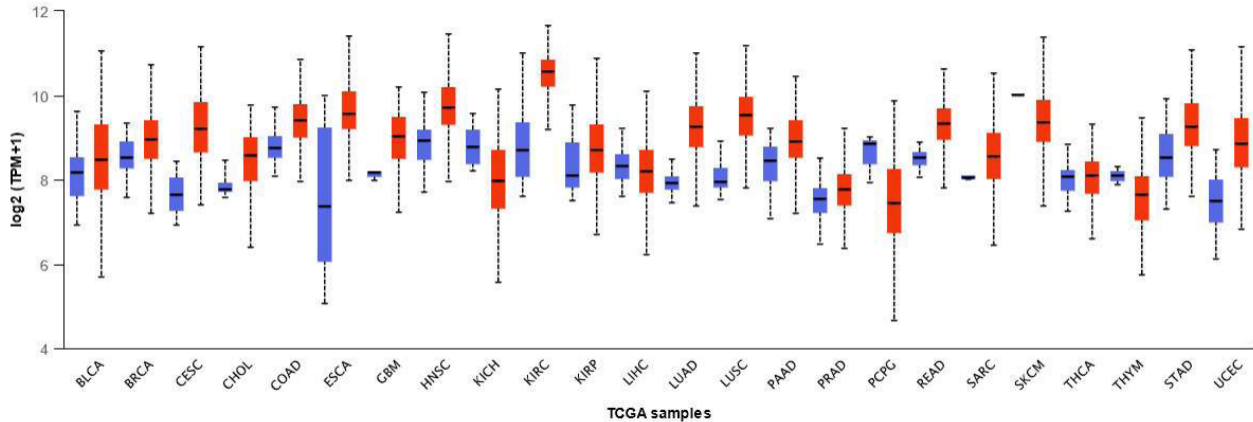

Supplement: Supplementary 2 — Figure S2: expression of LDHA across TCGA cancers (with tumor and normal samples) analyzed from http://ualcan.path.uab.edu. [file 9516774.f2.pdf]

Effect of LDHA expression level on PRAD patient survival

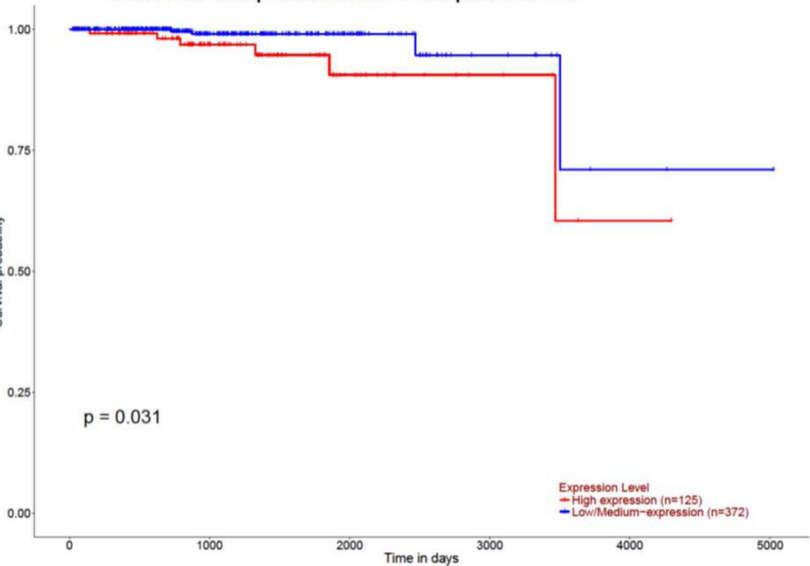

Supplement: Supplementary 3 — Figure S3: effect of LDHA expression level on PRAD patient survival analyzed by the Kaplan-Meier plotter from https://kmplot.com/analysis/. [file 9516774.f3.pdf]
